# Could Cardiovascular Health Metrics Account for Age and Sex Disparities in Self-Reported Ischemic Heart Disease Prevalence?

**DOI:** 10.3390/jcm7100369

**Published:** 2018-10-19

**Authors:** Yang Peng, Zhiqiang Wang

**Affiliations:** Centre for Chronic Disease, School of Clinical Medicine, The University of Queensland, Herston, QLD 4006, Australia; z.wang@uq.edu.au

**Keywords:** cardiovascular health, ischemic heart disease, Australian adults

## Abstract

The American Heart Association has outlined seven modifiable cardiovascular health (CVH) metrics. However, the sex and age disparities in the association between those CVH metrics and ischemic heart disease (IHD) prevalence are unclear. Our study sought to examine the possible sex and age variations in the association between CVH metrics and IHD prevalence using an Australian nationally representative survey. We used the core sample of the 2011–2012 Australian Health Survey, and 7499 adults with fasting plasma glucose (FPG) and total cholesterol values were included. We used Poisson regression analysis to measure the associations between individual metrics and IHD prevalence. Our study used both stratification and interaction analyses to compare the magnitude of associations between sex and age groups. Then, we calculated the population attributable fractions to measure the contribution of each metric to IHD prevalence. In addition, we applied logistic regression analysis to examine the influences of ideal CVH metrics number on IHD prevalence and used stratification and interaction analyses. Body mass index, physical activity, blood pressure, and FPG have greater effects on IHD prevalence in young adults compared to older adults. We failed to detect the sex variations in CVH metrics and IHD prevalence. The ideal CVH metrics number was inversely correlated to IHD prevalence and it has similar effects in four subgroups. These CVH metrics do not explain the sex and age disparities in IHD prevalence and the topic need further explorations.

## 1. Introduction

Ischemic heart disease (IHD) is one common type of cardiovascular disease (CVD) and is a significant contributor to deaths worldwide. According to the Global Burden of Disease Study, IHD led to nearly 10 million deaths worldwide in the year 2016, which accounted for 17.3% of all deaths [1]. There is evidence that a large proportion of IHD burden could be attributable to modifiable factors. A Chinese cohort found that almost half of the IHD cases could be prevented if the participants adhered to six healthy lifestyle factors, including smoking, alcohol consumption, physical activity, dietary pattern, and body mass index (BMI) [2]. In a US study, six lifestyle factors could explain 73% of coronary heart disease (CHD), another term for IHD, events in a 20-years follow-up period [3].

The American Heart Association (AHA) has outlined seven modifiable metrics [smoking, BMI, physical activity, dietary pattern, total cholesterol (TC), blood pressure (BP), and fasting plasma glucose (FPG)] to define and monitor cardiovascular health (CVH) status in the general population [4]. Some studies have reported that ideal status of those metrics played a crucial role in the reduction of IHD risk [5,6]. A few studies have suggested that the strengths of association between modifiable factors and IHD risk differed by age and sex [7,8] while the variations were inconclusive due to very limited studies.

In our study, we analysed and compared the strengths of association between CVH metrics and IHD prevalence in sex and age-specific subpopulations using a nationally representative survey in Australia.

## 2. Methods

### 2.1. Study Design and Participants

Details of the study design and participants have been described previously [5]. Briefly, our study used the core sample of 2011–2012 Australian Health Survey, which is a nationally representative health survey of the general Australians. Among 24,910 adults (≥18 years), we focused on those with available TC and FPG results, yielding to an overall sample size of 7499. Participants were classified into two age groups: young (<60 years) and older (≥60 years).

### 2.2. CVH Metrics

The definitions of the seven CVH metrics were described elsewhere [5]. Subjects with 0–2, 3–4, and 5–7 ideal metrics were regarded as having overall poor, intermediate, and ideal CVH status [9].

### 2.3. Outcome Measurement

The IHD prevalence was self-reported and based on the 10th version of International Classification of Diseases, codes I20–I25. To be more specific, participants were asked if they had been told by a doctor or nurse that they had IHD and they currently have IHD while taking the survey. They would be regarded as positive for IHD if they answered “yes” to both questions.

### 2.4. Covariates

The following variables were adjusted as covariates in our study: age, sex, education attainment, income status, and residence region. Education attainment was categorized as high (≥12 school years) and low (<12 school years). Income status was evaluated by household income and dichotomized as low (≤50th percentile equivalised weekly household income) and high (>50th percentile equivalised weekly household income). Residence region was classified into major cities, inner regional areas and other areas (outer regional and remote).

### 2.5. Statistical Analysis

Firstly, we compared the proportions of IHD, ideal metrics, and covariates between sex and age groups using weighted chi-square tests. 

Secondly, we explored the possible sex and age variations in the associations and contributions of individual metrics and IHD prevalence. We calculated crude (unadjusted) and adjusted incidence rate ratios (IRRs) and corresponding 95% confidence intervals (CIs), using univariate and multivariate Poisson regression analyses, to clarify the association between CVH metrics and IHD prevalence in each sex and age-specific group. We entered the interaction terms between sex/age and metrics in the adjusted models to explore the potential sex/age differences in the association.

Additionally, we calculated adjusted population attributable fractions (PAFs), for each sex and age group, based on the following equation to measure the effects of each metric on IHD reduction. *Pe* is the prevalence of unideal metric and Rate Ratios (RRs) were replaced with adjusted IRRs.

$$PAF=\frac{Pe\times \left(RR-1\right)}{1+Pe\times \left(RR-1\right)}$$

Thirdly, we calculated odds ratios (ORs) using logistic regression analyses to explore the relationship between overall CVH status categories and IHD prevalence in sex and age subgroups. Interaction terms between sex/age and CVH categories were included in the adjusted models. Participants with missing values in at least one CVH metrics were not included in the analyses.

To infer results for the in-scope population, we used biomedical weight and group Jackknife method with 60 replicate weights in the CVH metrics and IHD association analyses. All analyses were conducted within the Australian Bureau of Statistics (ABS)’s Remote Access Data Laboratory with Stata 10.0. A two-tailed *p*-value <0.05 was used to determine statistical significance.

## 3. Results

### 3.1. Baseline Characteristics

The basic characteristics of the included participants were displayed in Table 1. Overall, weighted 3.3% participants were positive for IHD prevalence (357/7499). Males have higher IHD prevalence than did the females (4.2% versus 2.5%, *p* < 0.01) and older adults have statistically higher IHD prevalence when compared to young adults (10.2% versus 1.1%, *p* < 0.01). Females had higher proportions of ideal smoking, BMI, dietary pattern, BP, and FPG and lower ideal physical activity proportion and income status than did males. They have similar ideal TC prevalence, education level, and regional distribution. Compared to young adults, older adults had a significantly higher percentage of ideal dietary pattern and lower prevalence of ideal status for the other six metrics. Young adults had higher income and education level and were more likely to reside in major cities as compared to older adults.

### 3.2. Sex and Age-Specific Effects of Individual Metrics on IHD Prevalence

Table 2 and Table 3 list the sex and age-specific crude and adjusted IRRs and 95% CIs by CVH metric for IHD prevalence, respectively. After adjusted for covariates, physical inactivity (IRR: 1.84, 95% CI: 1.00–3.39, *p* = 0.048) and elevated TC values (IRR: 1.53, 95% CI: 1.04–2.25, *p* = 0.03) were significant IHD risk factors for males. None of the metrics was associated with IHD prevalence for females. For the analyses of interaction terms, we failed to detect the sexual differences in the associations between those metrics and IHD prevalence (*P*_interaction_ > 0.05). Physical inactivity (IRR: 1.63, 95% CI: 1.01–2.64, *p* = 0.046) and elevated TC (IRR: 1.67, 95% CI: 1.11–2.51, *p* = 0.02) were significant IHD risk factors for older adults. None of the metrics was associated with IHD prevalence for young adults. The effects of high BMI (*P*_interaction_ = 0.01), physical inactivity (*P*_interaction_ = 0.03), elevated BP (*P*_interaction_ <0.01), and elevated FPG (*P*_interaction_ = 0.04) were more pronounced among young adults than in older adults.

**Figure 1:**
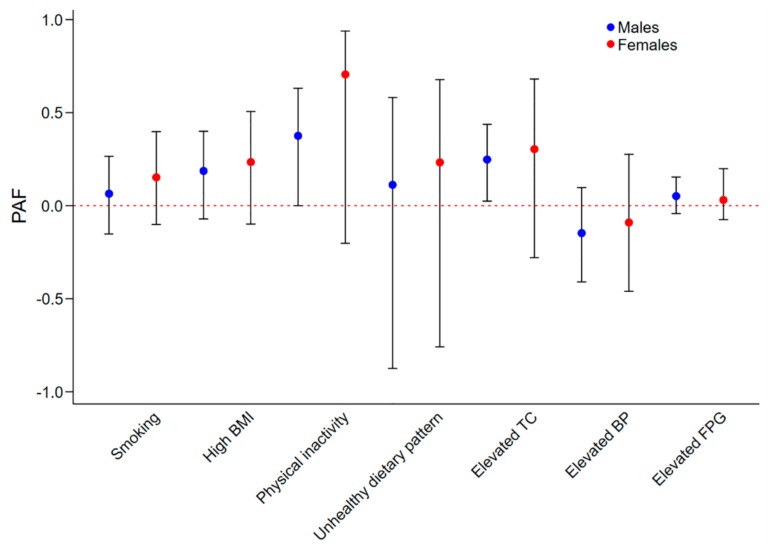
Adjusted PAFs of CVH metrics to IHD prevalence, stratified by sex. Abbreviations: PAF, population attributable fraction; TC, total cholesterol; BP, blood pressure; FPG, fasting plasma glucose; CVH, cardiovascular health; IHD, ischemic heart disease.

**Figure 2:**
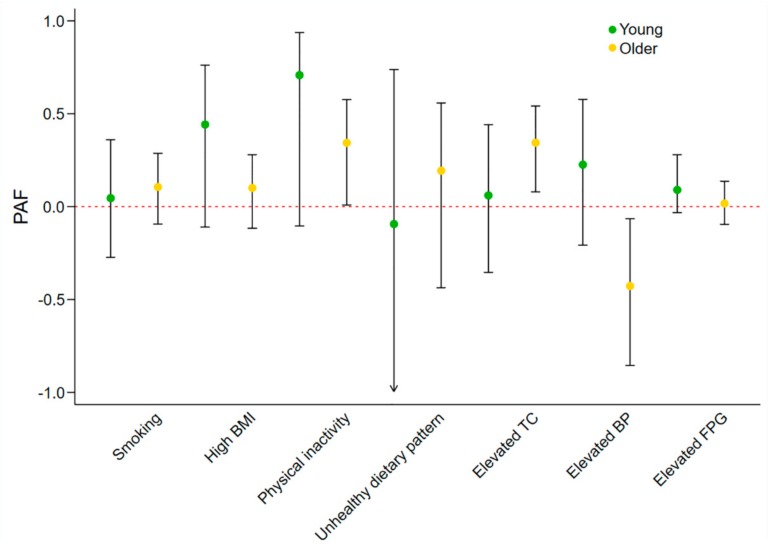
Adjusted PAFs of CVH metrics to IHD prevalence, stratified by age. Abbreviations: PAF, population attributable fraction; TC, total cholesterol; BP, blood pressure; FPG, fasting plasma glucose; CVH, cardiovascular health; IHD, ischemic heart disease.

The sex and age-specific adjusted PAFs were displayed in Figure 1 and Figure 2, respectively. Among males, physical inactivity and elevated TC contributed to 37.6% and 24.8% of IHD burden (Figure 1). Elevated TC and physical inactivity contributed to 34.4% and 34.3% of IHD burden in older adults (Figure 2). None of the seven metrics was significant contributor of IHD in females and younger adults.

### 3.3. Sex and Age-Specific Effects of Number of Ideal CVH Metrics on IHD Prevalence

We explored the association between number of ideal CVH metrics and IHD prevalence based on 7002 participants who have no missing data on the seven metrics and the crude and adjusted ORs were shown in Table 4. We observed a negative association between the number of ideal CVH metrics and IHD prevalence in all sex and age subpopulations. Among females and young adults, we noticed a 59% (OR, 0.41; 95% CI, 0.19–0.88) and a 65% (OR, 0.35; 95% CI, 0.15–0.83) reductions in the odds of IHD prevalence for those with intermediate CVH status and none of those with ideal CVH status had self-reported IHD. For males and older adults, we observed non-significant reductions in the odds of IHD prevalence for those with intermediate and ideal CVH. In addition, one more ideal CVH metric was associated with 11–31% reduced IHD prevalence while the association failed to reach significant level for males and older adults. The analyses of interaction terms indicated that the effects of overall CVH status on IHD prevalence were similar among the four subgroups.

## 4. Discussion

By analyzing the nationally representative survey, we did not reveal the sexual variations in the strength of seven metrics and IHD prevalence. Our results demonstrated that the impacts of high BMI, physical inactivity, elevated BP, and elevated FPG were more apparent in young adults than in older adults. The association between ideal metrics number and IHD prevalence are similar among the four subgroups.

Although we found physical inactivity and elevated TC were significant IHD risk factors and contributors for only males, we did not reveal the differences in the IHD association strengths for all the metrics between men and women. However, a few recent meta-analyses have suggested the possible sex disparities in the effect sizes between CVH metrics and IHD [10,11,12]. It appears that smoking and diabetes have a more potent influence on CHD risk in women [10,11] whereas elevated TC has more adverse effect in men [12]. We observed that physical inactivity and elevated TC were significant IHD risk factors for older adults instead of young adults. While the findings of interaction terms did not further support the possible age variations. Instead, the interaction term analyses implied the increased BMI, physical inactivity, elevated BP and elevated FPG are associated with greater prevalence in young adults than older adults are. To our knowledge, no meta-analysis explored the age disparities between CVH metrics and IHD risk. The decreasing effects of high BMI [13], elevated BP [13,14], and elevated FPG [13,15] with age were also noticed in a few observational studies. The age variations in the physical inactivity-IHD relationship were unclear. However, a study has noted the association between physical inactivity and overall CVD was stronger among young adults [16]. Given the inconclusive findings, more large-scale prospective studies are warranted.

We observed that increasing number of ideal metrics was associated with reduced IHD prevalence and the negative association was also found in other studies [2,6]. The very few IHD cases in those with ideal CVH status, especially in females and young adults, suggested the apparent protection effect of CVH status. The strengths of association was similar among the four groups, which indicate the disparities in IHD prevalence was less likely to be explained by the CVH metrics. A number of other factors, including education [17], income [18], and knowledge/awareness of IHD [19] were found to be associated with IHD prevalence and whether they could explain the sex and age disparities in IHD prevalence still need further explorations.

Our study has several strengths: we firstly explored sex and age variances in the associations between CVH metrics and IHD prevalence in Australian adults; we drawn our conclusions based on a nationally representative sample; we adjusted some factors that could be potential confounders. Our study also has several limitations. Firstly, the IHD status was self-reported and it may lead to overestimation or underestimation of IHD prevalence. Previous studies have explored the accuracy of self-reported myocardial infarction, a common type of IHD, and they observed moderate [20,21] or substantial [22,23] agreement between self-reported questionnaire and medical record data. We need studies with medical record diagnosed IHD to focus on the topic. Secondly, demographic factors, smoking, physical activity, and dietary pattern were self-reported, and they may not be wholly accurate. Thirdly, we could not explore the sex and age variations in the associations between CVH metrics and IHD incidence or mortality as our study was a cross-sectional study. Fourthly, we may still suffer from the selection bias even though, as recommended by the ABS, we applied biomedical weights.

## 5. Conclusions

In summary, we noticed the effects of high BMI, physical inactivity, elevated BP, and elevated FPG on IHD prevalence were greater in young adults relative to that in older adults. No sex differences were observed between individual CVH metrics and IHD prevalence. The impacts of ideal metrics number on IHD prevalence were similar among the four subgroups. Further large-scale prospective studies are warranted to confirm the possible sex and age disparities. 

## Figures and Tables

**Table 1:** Baseline characteristics of the included participants.

Metrics	Status	Males, *n* (%)	Females, *n* (%)	*p*	Young, *n* (%)	Older, *n* (%)	*p*
IHD	Yes	214 (4.2)	143 (2.5)	<0.01	72 (1.1)	285 (10.2)	<0.01
	No	3115 (95.8)	4027 (97.5)		4751 (98.9)	2391 (89.8)	
Smoking	Ideal	1420 (49.8)	2342 (61.1)	<0.01	2521 (57.7)	1241 (49.0)	<0.01
	Non-ideal	1909 (50.2)	1828 (38.9)		2302 (42.3)	1435 (51.0)	
BMI	Ideal	833 (32.3)	1562 (46.2)	<0.01	1750 (43.1)	645 (27.2)	<0.01
	Non-ideal	2388 (67.7)	2344 (53.8)		2843 (56.9)	1889 (72.8)	
Physical activity	Ideal	945 (32.7)	829 (20.9)	<0.01	1320 (30.0)	454 (16.6)	<0.01
	Non-ideal	2382 (67.3)	3339 (79.1)		3500 (70.0)	2221 (83.4)	
Dietary pattern	Ideal	93 (2.1)	341 (7.4)	<0.01	223 (4.1)	211 (6.9)	<0.01
	Non-ideal	3236 (97.9)	3829 (92.6)		4600 (95.9)	2465 (93.1)	
TC	Ideal	1259 (45.1)	1568 (45.8)	0.70	2246 (52.5)	581 (23.9)	<0.01
	Non-ideal	2070 (54.9)	2602 (54.2)		2577 (47.5)	2095 (76.1)	
BP	Ideal	1047 (36.7)	1781 (51.6)	<0.01	2234 (51.4)	594 (21.9)	<0.01
	Non-ideal	2189 (63.3)	2204 (48.4)		2427 (48.6)	1966 (78.1)	
FPG	Ideal	2426 (79.4)	3513 (87.7)	<0.01	4162 (88.6)	1777 (68.4)	<0.01
	Non-ideal	903 (20.6)	657 (12.3)		661 (11.4)	899 (31.6)	
Age	<60 years	2078 (76.2)	2745 (74.7)	0.01	4823 (100.0)	0 (0.0)	NA
	≥60 years	1251 (23.8)	1425 (25.3)		0 (0.0)	2676 (100.0)	
Sex	Male	3329 (100.0)	0 (0.0)	NA	2078 (49.8)	1251 (47.8)	0.01
	Female	0 (0.0)	4170 (100.0)		2745 (50.2)	1425 (52.2)	
Education level	High	1620 (57.6)	2113 (58.1)	0.76	2969 (67.5)	764 (28.3)	<0.01
	Low	1709 (42.4)	2057 (41.9)		1854 (32.5)	1912 (71.7)	
Income	High	1642 (56.5)	1758 (49.9)	<0.01	2771 (62.0)	629 (26.6)	<0.01
	Low	1441 (43.5)	1976 (50.1)		1628 (38.0)	1789 (73.4)	
Region	Major cities	2024 (72.0)	2538 (73.0)	0.71	3012 (74.2)	1550 (67.2)	<0.01
	Inner regional	760 (20.0)	949 (19.2)		1025 (18.3)	684 (23.7)	
	Other	545 (8.0)	683 (7.8)		786 (7.5)	442 (9.1)	

Numbers and percentages were expressed as weighted and unweighted and *p* values were from weighted chi-square tests. Abbreviations: IHD, ischemic heart disease; BMI, body mass index; TC, total cholesterol; BP, blood pressure; FPG, fasting plasma glucose; NA, not applicable.

**Table 2:** IRRs between individual CVH metrics and IHD prevalence, stratified by sex.

Metrics	Population	Crude IRR (95% CI)	*p*	Adjusted IRR * (95% CI)	*p*	*P*_interaction_
Smoking	Males	1.84 (1.21–2.79)	0.01	1.12 (0.77–1.63)	0.56	0.49
	Females	1.30 (0.78–2.18)	0.31	1.41 (0.79–2.51)	0.24	
High BMI	Males	1.93 (1.26–2.97)	<0.01	1.32 (0.91–1.90)	0.14	0.14
	Females	2.13 (1.27–3.58)	0.01	1.51 (0.85–2.71)	0.15	
Physical inactivity	Males	3.57 (1.94–6.55)	<0.01	1.84 (1.00–3.39)	0.048	0.38
	Females	9.47 (1.88–47.73)	0.01	3.99 (0.79–20.12)	0.09	
Unhealthy dietary pattern	Males	0.42 (0.17–1.06)	0.06	1.13 (0.52–2.43)	0.76	0.82
	Females	1.24 (0.50–3.07)	0.64	1.33 (0.53–3.29)	0.54	
Elevated TC	Males	2.56 (1.66–3.95)	<0.01	1.53 (1.04–2.25)	0.03	0.85
	Females	5.22 (2.07–13.18)	<0.01	1.70 (0.65–4.42)	0.28	
Elevated BP	Males	1.62 (1.07–2.45)	0.02	0.81 (0.57–1.16)	0.25	0.89
	Females	3.46 (1.93–6.21)	<0.01	0.85 (0.43–1.69)	0.63	
Elevated FPG	Males	2.56 (1.84–3.55)	<0.01	1.20 (0.85–1.67)	0.29	1.00
	Females	2.98 (1.66–5.34)	<0.01	1.20 (0.56–2.57)	0.64	

* Adjusted for age, education attainment, income, and residence region. Abbreviations: IRR, incidence rate ratio; CI, confidence interval; BMI, body mass index; TC, total cholesterol; BP, blood pressure; FPG, fasting plasma glucose.

**Table 3:** IRRs between individual CVH metrics and IHD prevalence, stratified by age.

Metrics	Population	Crude IRR (95% CI)	*p*	Adjusted IRR * (95% CI)	*p*	*P*_interaction_
Smoking	Young adults	1.67 (0.89–3.14)	0.11	1.10 (0.55–2.18)	0.78	0.48
	Older adults	1.35 (0.95–1.92)	0.09	1.22 (0.84–1.75)	0.29	
High BMI	Young adults	4.42 (1.64–11.92)	<0.01	2.28 (0.84–6.16)	0.10	0.01
	Older adults	1.07 (0.78–1.47)	0.66	1.15 (0.86–1.52)	0.34	
Physical inactivity	Young adults	7.08 (1.56–32.16)	0.01	4.34 (0.87–21.55)	0.07	0.03
	Older adults	1.91 (1.17–3.12)	0.01	1.63 (1.01–2.64)	0.046	
Unhealthy dietary pattern	Young adults	0.93 (0.21–4.11)	0.93	0.91 (0.21–3.95)	0.90	0.31
	Older adults	1.27 (0.66–2.44)	0.47	1.26 (0.67–2.37)	0.47	
Elevated TC	Young adults	2.69 (1.27–5.66)	0.01	1.12 (0.51–2.48)	0.77	0.51
	Older adults	1.47 (0.98–2.21)	0.06	1.67 (1.11–2.51)	0.02	
Elevated BP	Young adults	3.69 (1.92–7.12)	<0.01	1.56 (0.67–3.62)	0.29	<0.01
	Older adults	0.78 (0.50–1.22)	0.28	0.61 (0.40–0.92)	0.02	
Elevated FPG	Young adults	3.60 (1.77–7.30)	<0.01	1.72 (0.77–3.83)	0.18	0.04
	Older adults	1.29 (0.92–1.80)	0.14	1.05 (0.74–1.47)	0.79	

* Adjusted for age, sex, education attainment, income, and residence region. Abbreviations: IRR, incidence rate ratio; CI, confidence interval; BMI, body mass index; TC, total cholesterol; BP, blood pressure; FPG, fasting plasma glucose.

**Table 4:** ORs between number of ideal metrics and IHD prevalence, stratified by sex and age.

Participants	Ideal Metrics Number	IHD Cases/Participants	Crude OR (95% CI)	*p*	Adjusted * OR (95% CI)	*p*
Males	0–2	150/1741	Reference	NA	Reference	NA
	3–4	44/1147	0.34 (0.21–0.56)	<0.01	0.65 (0.42–1.01)	0.06
	5–7	4/285	0.06 (0.01–0.51)	0.01	0.47 (0.05–4.30)	0.50
	One more ideal metric	NA	0.61 (0.53–0.70)	<0.01	0.86 (0.73–1.02)	0.09
Females	0–2	99/1601	Reference	NA	Reference	NA
	3–4	29/1639	0.22 (0.11–0.45)	<0.01	0.41 (0.19–0.88)	0.02
	5–7	0/589	NA	NA	NA	NA
	One more ideal metric	NA	0.52 (0.45–0.60)	<0.01	0.73 (0.59–0.91)	0.01
Young	0–2	54/1708	Reference	NA	Reference	NA
	3–4	15/2028	0.18 (0.09–0.37)	<0.01	0.35 (0.15–0.83)	0.02
	5–7	0/792	NA	NA	NA	NA
	One more ideal metric	NA	0.49 (0.40–0.60)	<0.01	0.69 (0.52–0.91)	0.01
Older	0–2	195/1634	Reference	NA	Reference	NA
	3–4	58/758	0.61 (0.39–0.95)	0.03	0.68 (0.44–1.03)	0.07
	5–7	4/82	0.35 (0.04–2.91)	0.32	0.55 (0.06–5.09)	0.59
	One more ideal metric	NA	0.83 (0.71–0.97)	0.02	0.89 (0.76–1.05)	0.17

* Adjusted for age, education attainment, income, and residence region for sex-specific population and additionally adjusted for sex for age-specific population. Abbreviations: OR, odds ratio; IHD, ischemic heart disease; CI, confidence interval; NA, not applicable.

## References

[1] GBD 2016 Causes of Death Collaborators (2017). Global, regional, and national age-sex specific mortality for 264 causes of death, 1980–2016: A systematic analysis for the Global Burden of Disease Study 2016. Lancet.

[2] Lv J., Yu C., Guo Y., Bian Z., Yang L., Chen Y., Tang X., Zhang W., Qian Y., Huang Y. (2017). Adherence to Healthy Lifestyle and Cardiovascular Diseases in the Chinese Population. J. Am. Coll. Cardiol..

[3] Chomistek A.K., Chiuve S.E., Eliassen A.H., Mukamal K.J., Willett W.C., Rimm E.B. (2015). Healthy lifestyle in the primordial prevention of cardiovascular disease among young women. J. Am. Coll. Cardiol..

[4] Lloyd-Jones D.M., Hong Y., Labarthe D., Mozaffarian D., Appel L.J., Van Horn L., Greenlund K., Daniels S., Nichol G., Tomaselli G.F. (2010). Defining and setting national goals for cardiovascular health promotion and disease reduction: The American Heart Association’s strategic Impact Goal through 2020 and beyond. Circulation.

[5] Peng Y., Wang Z., Dong B., Cao S., Hu J., Adegbija O. (2017). Life’s Simple 7 and ischemic heart disease in the general Australian population. PLoS ONE.

[6] Yang Q., Cogswell M.E., Dana Flanders W., Hong Y., Zhang Z., Loustalot F., Gillespie C., Merritt R., Hu F.B. (2012). Trends in cardiovascular health metrics and associations with all-cause and CVD mortality among us adults. JAMA.

[7] Hackshaw A., Morris J.K., Boniface S., Tang J.L., Milenkovic D. (2018). Low cigarette consumption and risk of coronary heart disease and stroke: Meta-analysis of 141 cohort studies in 55 study reports. BMJ.

[8] Huxley R.R., Hirakawa Y., Hussain M.A., Aekplakorn W., Wang X., Peters S.A., Mamun A., Woodward M. (2015). Age- and Sex-Specific Burden of Cardiovascular Disease Attributable to 5 Major and Modifiable Risk Factors in 10 Asian Countries of the Western Pacific Region. Circ. J..

[9] Gaye B., Canonico M., Perier M.C., Samieri C., Berr C., Dartigues J.F., Tzourio C., Elbaz A., Empana J.P. (2017). Ideal Cardiovascular Health, Mortality, and Vascular Events in Elderly Subjects: The Three-City Study. J. Am. Coll. Cardiol..

[10] Huxley R., Barzi F., Woodward M. (2006). Excess risk of fatal coronary heart disease associated with diabetes in men and women: Meta-analysis of 37 prospective cohort studies. BMJ.

[11] Huxley R.R., Woodward M. (2011). Cigarette smoking as a risk factor for coronary heart disease in women compared with men: A systematic review and meta-analysis of prospective cohort studies. Lancet.

[12] Peters S.A., Singhateh Y., Mackay D., Huxley R.R., Woodward M. (2016). Total cholesterol as a risk factor for coronary heart disease and stroke in women compared with men: A systematic review and meta-analysis. Atherosclerosis.

[13] Singh G.M., Danaei G., Farzadfar F., Stevens G.A., Woodward M., Wormser D., Kaptoge S., Whitlock G., Qiao Q., Lewington S. (2013). The age-specific quantitative effects of metabolic risk factors on cardiovascular diseases and diabetes: A pooled analysis. PLoS ONE.

[14] Lacey B., Lewington S., Clarke R., Kong X.L., Chen Y., Guo Y., Yang L., Bennett D., Bragg F., Bian Z. (2018). Age-specific association between blood pressure and vascular and non-vascular chronic diseases in 0.5 million adults in China: A prospective cohort study. Lancet Glob. Health.

[15] Rao Kondapally Seshasai S., Kaptoge S., Thompson A., Di Angelantonio E., Gao P., Sarwar N., Whincup P.H., Mukamal K.J., Gillum R.F., Holme I. (2011). Diabetes mellitus, fasting glucose, and risk of cause-specific death. N. Engl. J. Med..

[16] Manson J.E., Greenland P., LaCroix A.Z., Stefanick M.L., Mouton C.P., Oberman A., Perri M.G., Sheps D.S., Pettinger M.B., Siscovick D.S. (2002). Walking compared with vigorous exercise for the prevention of cardiovascular events in women. N. Engl. J. Med..

[17] Tillmann T., Vaucher J., Okbay A., Pikhart H., Peasey A., Kubinova R., Pajak A., Tamosiunas A., Malyutina S., Hartwig F.P. (2017). Education and coronary heart disease: Mendelian randomisation study. BMJ.

[18] Konttinen H., Kilpi F., Moustgaard H., Martikainen P. (2016). Socioeconomic Position and Antidepressant Use as Predictors of Coronary Heart Disease Mortality: A Population-Based Registry Study of 362,271 Finns. Psychosom. Med..

[19] Ramachandran H.J., Wu V.X., He H.G., Jiang Y., Wang W. (2016). Awareness, knowledge, healthy lifestyle behaviors, and their correlates to coronary heart disease among working women in Singapore. Heart Lung.

[20] Yasaitis L.C., Berkman L.F., Chandra A. (2015). Comparison of self-reported and Medicare claims-identified acute myocardial infarction. Circulation.

[21] Muggah E., Graves E., Bennett C., Manuel D.G. (2013). Ascertainment of chronic diseases using population health data: A comparison of health administrative data and patient self-report. BMC Public Health.

[22] Okura Y., Urban L.H., Mahoney D.W., Jacobsen S.J., Rodeheffer R.J. (2004). Agreement between self-report questionnaires and medical record data was substantial for diabetes, hypertension, myocardial infarction and stroke but not for heart failure. J. Clin. Epidemiol..

[23] Bolland M.J., Barber A., Doughty R.N., Grey A., Gamble G., Reid I.R. (2013). Differences between self-reported and verified adverse cardiovascular events in a randomised clinical trial. BMJ Open.

